# Supplementary material for: Excitatory and inhibitory synapse reorganization immediately after critical sensory experience in a vocal learner
Source: eLife. 2018 Oct 25;7:e37571. doi: 10.7554/eLife.37571 (PMC6255392; doi:10.7554/eLife.37571)
Supplement: Transparent reporting form [file elife-37571-transrepform.docx]

***eLife’s* transparent reporting form**

We encourage authors to provide detailed information *within their submission* to facilitate the interpretation and replication of experiments. Authors can upload supporting documentation to indicate the use of appropriate reporting guidelines for health-related research (see [EQUATOR Network](http://www.equator-network.org/%20)), life science research (see the [BioSharing Information Resource](https://biosharing.org/)), or the [ARRIVE guidelines](http://www.plosbiology.org/article/info:doi/10.1371/journal.pbio.1000412) for reporting work involving animal research. Where applicable, authors should refer to any relevant reporting standards documents in this form.

If you have any questions, please consult our Journal Policies and/or contact us: [editorial@elifesciences.org](mailto:editorial@elifesciences.org).

**Sample-size estimation**

- You should state whether an appropriate sample size was computed when the study was being designed
- You should state the statistical method of sample size computation and any required assumptions
- If no explicit power analysis was used, you should describe how you decided what sample (replicate) size (number) to use

Please outline where this information can be found within the submission (e.g., sections or figure legends), or explain why this information doesn’t apply to your submission:

We performed a Group Sequential Design. The sample size (n=4 animals in each group and n>100 synapses per animal) was motivated by our choice of using linear mixed effects models for significance testing. Note that our sample size is larger than that in many electron microscopy investigations (where typically 1-3 animals are used per group). To perform a population-level analysis, we performed a biological replicate of one of the group (ISO group).

**Replicates**

- You should report how often each experiment was performed
- You should include a definition of biological versus technical replication
- The data obtained should be provided and sufficient information should be provided to indicate the number of independent biological and/or technical replicates
- If you encountered any outliers, you should describe how these were handled
- Criteria for exclusion/inclusion of data should be clearly stated
- High-throughput sequence data should be uploaded before submission, with a private link for reviewers provided (these are available from both GEO and ArrayExpress)

Please outline where this information can be found within the submission (e.g., sections or figure legends), or explain why this information doesn’t apply to your submission:

Each group contained four animals. For the ISO group, we performed a biological replicate (ISO60 group). We did not perform technical replicates. We provide access to all data and did not remove any outliers. In the Section FIBSEM Synapse Segmentation, we excluded objects smaller than 1000 voxels, because they were too small to be synapses.

**Statistical reporting**

- Statistical analysis methods should be described and justified
- Raw data should be presented in figures whenever informative to do so (typically when N per group is less than 10)
- For each experiment, you should identify the statistical tests used, exact values of N, definitions of center, methods of multiple test correction, and dispersion and precision measures (e.g., mean, median, SD, SEM, confidence intervals; and, for the major substantive results, a measure of effect size (e.g., Pearson's r, Cohen's d)
- Report exact p-values wherever possible alongside the summary statistics and 95% confidence intervals. These should be reported for all key questions and not only when the p-value is less than 0.05.

Please outline where this information can be found within the submission (e.g., sections or figure legends), or explain why this information doesn’t apply to your submission:

For the synapse data, we did a linear mixed-effect analysis, because our data are non-independent and hierarchical (sections per bird in a given group compared to other groups). For estimation of percent symmetric synapses, we did both 1) a linear mixed-effect analysis by grouping the disectors into 4 non-overlapping groups and 2) a jackknife resampling procedure (because many disectors contained no dissected synapses) followed by a bootstrapping procedure. Both approaches yielded similarly high statistical significance.

For each group, we show the means in individual birds ± the standard deviations.

The exact p value was given in all cases, except in the Synapse size section.

(For large datasets, or papers with a very large number of statistical tests, you may upload a single table file with tests, Ns, etc., with reference to sections in the manuscript.)

**Group allocation**

- Indicate how samples were allocated into experimental groups (in the case of clinical studies, please specify allocation to treatment method); if randomization was used, please also state if restricted randomization was applied
- Indicate if masking was used during group allocation, data collection and/or data analysis

Please outline where this information can be found within the submission (e.g., sections or figure legends), or explain why this information doesn’t apply to your submission:

We wrote in Experimental procedures, “to avoid human biases, we coded all birds with numeric identifiers and performed all analyses in a manner that was blind to bird identity and treatment group.”

**Additional data files (“source data”)**

- We encourage you to upload relevant additional data files, such as numerical data that are represented as a graph in a figure, or as a summary table
- Where provided, these should be in the most useful format, and they can be uploaded as “Source data” files linked to a main figure or table
- Include model definition files including the full list of parameters used
- Include code used for data analysis (e.g., R, MatLab)
- Avoid stating that data files are “available upon request”

Please indicate the figures or tables for which source data files have been provided:

We provide all SSEM synapse density data for Experiments I and II in the Matlab file ssSEM_exp1and2_groupSeperated.mat. We provide all FIBSEM data for Experiment I in the Matlab file FIBSEM_exp1.mat. HVC volume data for Experiment II is provided in the Matlab file HVCvolume_exp2.mat.

To reproduce our linear mixed effects analyses, we provide the Matlab function getLME. For example, to reproduce the comparison between synaptic densities in LONG and LONG60 birds, one first needs to load the data: *load ssSEM_exp1and2_groupSeperated*, then one needs to concatenate the relevant variables: *data=vertcat(data_ssSEM_exp1_LONG,data_ssSEM_exp2_TUT60),* and finally, one needs to run the function:  *getLME(data),* followed by typing *1* for running the analysis for asymmetric synapses.

All Matlab files can be retrieved from <https://www.research-collection.ethz.ch/handle/20.500.11850/285394>, DOI 10.3929/ethz-b-000285394.
